# Impaired immune reconstitution in HIV infection: the role of CD4^+^ T-cell-associated NKG2D ligands, CD4^+^ T-cell subsets imbalance, and immune function deficiency

**DOI:** 10.3389/fimmu.2025.1541574

**Published:** 2025-02-21

**Authors:** Qianqian Xu, Qiuyue Zhang, Peng Xu, Tong Zhang, Hao Wu, Xin Zhang, Christiane Moog, Bin Su

**Affiliations:** ^1^ Beijing Key Laboratory for HIV/AIDS Research, Clinical and Research Center for Infectious Diseases, Beijing Youan Hospital, Capital Medical University, Beijing, China; ^2^ Sino-French Joint Laboratory for HIV/AIDS Research, Sino-French Joint Laboratory for Research on Humoral Immune Response to HIV Infection, Beijing Youan Hospital, Capital Medical University, Beijing, China; ^3^ Department of Immunity and Infectious Diseases, Third People’s Hospital of Shenzhen, National Clinical Research Center for Infectious Diseases, Shenzhen, Guangdong, China; ^4^ Laboratoire d’ImmunoRhumatologie Moléculaire, Institut National de la Santé et de la Recherche Médicale (INSERM) UMR_S 1109, Institut Thématique Interdisciplinaire (ITI) de Médecine de Précision de Strasbourg, Transplantex NG, Faculté de Médecine, Fédération Hospitalo-Universitaire OMICARE, Fédération de Médecine Translationnelle de Strasbourg (FMTS), Université de Strasbourg, Strasbourg, France

**Keywords:** immunological non-responders, NK cell ligands, CD4+T cell subsets, HIV, aids, NKG2D

## Abstract

**Objective:**

The role of natural killer (NK) cells, which mediate innate immunity, in the immune reconstitution of people living with HIV (PLWH) remains unclear. Our previous research indicated that early activation of CD56^dim^CD16^dim/-^ NK cells plays an important role in the recovery of CD4^+^ T cells in immunological non-responders (INRs) after ART. This study mainly focuses on the profiles of cell receptors and their relative ligands for NK cells and CD4^+^ T cells exhibited on INRs and immunological responders (IRs) in order to analyze the impact of differential immune status on immune reconstitution in PLWH receiving ART.

**Methods:**

This study included 66 PLWH who had been on ART for 4 years, comprising 32 INRs and 34 IRs. Using flow cytometry, we examined the expression of cell receptors and ligands for NK cells and CD4^+^ T cells in PBMCs, as well as the differentiation of CD4^+^ T cells.

**Results:**

The expression of NKG2D ligands, including MICA/B and ULBP2-5, on CD4^+^ T cells in INRs is elevated prior to ART. Further research found that the expression of CD95 on MICA/B^+^CD4^+^ T cells and ULBP2-5^+^CD4^+^ T cells was higher in INRs before ART compared to IRs. Simultaneously, the percentages of death receptor CD95 expression on MICA/B^+^CD4^+^ T cells and on ULBP2-5^+^CD4^+^ T cells before ART were negatively correlated with CD4^+^ T-cell counts and ΔCD4. Among the CD4^+^ T-cell subsets, an imbalance persists in the CD4^+^ Tcm and CD4^+^ Temra subsets in both INRs and IRs, before or after ART. CD4^+^ T cells exhibit elevated levels of activation, proliferation, exhaustion, and apoptosis prior to ART initiation. However, CD4^+^ T-cell activation and proliferation normalize post-ART, while apoptosis and exhaustion levels remain significantly elevated. Regardless of ART, the anti-apoptotic capacity of CD4+ T cells in INRs is still lower than that of IRs and healthy controls (HCs). Before ART, the frequency of CD31 expression on naive CD4^+^ T cells in INRs is lower than that in IRs and HCs. Following ART, the amounts of CD31^+^ Tn from CD4^+^ T cells remain impaired in both INRs and IRs compared to HCs.

**Conclusion:**

The upregulation of related ligands for the NKG2D receptor on CD4^+^ T cells in INRs is associated with increased susceptibility of CD4^+^ T cells to NK cell-mediated killing. CD95 may plays an important role in poor recovery of CD4^+^ T cells co-expressing NKG2D-related ligands. The imbalance in CD4^+^ Tcm and CD4^+^ Temra subset homeostasis and impaired CD31 expression on naive CD4^+^ T cells in INRs are associated with poor immune reconstitution outcomes.

## Introduction

AIDS, resulting from human immunodeficiency virus (HIV) infection, continues to present a significant global public health challenge. ART is highly effective in inhibiting HIV viral replication, increasing CD4^+^ T-cell counts in peripheral blood, recovery patients’ immune function, and reducing AIDS-related morbidity and mortality. However, despite these advancements, 10%~40% of people living with HIV (PLWH) undergoing ART do not achieve adequate CD4^+^ T-cell recovery and suffer from poor immune reconstitution (1, 2). These individuals are classified as immunological non-responders (INRs) (3, 4). In addition to being more susceptible to opportunistic infections, INRs experience higher rates of long-term morbidity and mortality compared to those who achieve complete immune recovery, known as immunological responders (IRs) (4). INRs face impaired immune function and accelerated immune senescence (5).

There has been limited prior research on the impact of the natural immune system’s failure on the immunological reconstitution of PLWH. Specifically, the role of natural killer (NK) cells, which are crucial effector cells mediating natural immunity, in contributing to immune reconstitution remains unclear. As natural immune cells, NK cells are essential for early antiviral and anti-tumor immunity (6–9). Following HIV infection, NK cells exert control over the disease by lysing HIV-infected cells and secreting chemokines that involved in mediating specific immune responses and blocking the CCR5 receptor on HIV-bound cells (10). However, virus-induced immune activation negatively impacts the distribution and function of NK cell subsets (7, 11).

NKG2D, an activating receptor for NK cells, is crucial for their activation and effector functions. In the diagnosis and treatment of autoimmune diseases, the inappropriate expression of NKG2D and its ligands have been implicated in disease pathogenesis (12). Meanwhile, the binding of NKG2D to its ligands activates NK cells, leading to cytokine production and the lysis of target cells, demonstrating potential in cancer therapy (13, 14). Furthermore, various cancer treatment modalities indirectly influence NKG2D ligands (15). For instance, in the treatment of multiple myeloma, modulating the NKG2D/NKG2DL axis can enhance the anti-tumor potential of immune cells (13). NKG2D recognizes a variety of ligands and exhibits differential affinities. In humans, NKG2D primarily recognizes MHC class I chain-related molecules from the A and B families (MICA and MICB) as well as six UL16-binding proteins (ULBP1-6) (16). Normal human cells typically do not express NKG2D ligands; however, their expression can be induced by tumors and viral infections (17, 18). Immune disruption and homeostatic imbalances further upregulate NKG2D ligand expression (17). Significant progress has been made in the therapeutic targeting of NKG2D receptor activation for the treatment of malignancies (14, 18). However, the aberrant upregulation of NKG2D ligands on the surface of T cells can lead to T-cell destruction, impaired immune function, and even immune exhaustion (19). Some studies have found that HIV upregulates NKG2D ligand expression including ULBP-1, -2, and -3, but not MICA or MICB, in infected cells. Vpr upregulates ULBP-2 expression on CD4^+^ T cells and promotes NK cell-mediated killing (20–22). This phenomenon may underlie the persistent failure of T-cell recovery observed in INRs.

Our previous research revealed that the inadequate recovery of CD4^+^ T-cell counts in INRs following ART was significantly influenced by NK cells, as indicated by the increased activation of NK cells and the rise in the number of CD56^dim^CD16^dim/-^ NK cells and fewer NKG2D expression on NK cells, in particular the subset of CD56^dim^CD16^dim/-^ NK cells (23). Furthermore, the distribution characteristics, functions, and activation status of NK cell and CD4^+^ T-cell subsets were found to play a crucial role during the ART process. A more comprehensive investigation into the mechanisms underlying the role of NK cells, CD4^+^ T-cell subsets in the immune reconstitution of PLWH is of great importance for informing the application of NK cell therapy in the treatment of HIV-1 infection.

It is well known that elevated expression of cell death receptors induces increased apoptosis. The death receptor family mainly consists of tumor necrosis factor receptor-1 (TNFR1), CD95 (also known as Fas and APO-1), and TRAIL receptors (TRAIL receptor-1, also known as DR4, and TRAIL receptor-2) (24). As members of the tumor necrosis factor receptor family, they play a key role in fighting tumors and maintaining homeostasis in the internal environment. Based on this, the present study hypothesizes that the aberrant expression of ligands recognized by NK cell receptors on CD4^+^ T cells and CD4^+^ T-cell apoptosis receptor expression is associated with upregulation of NKG2D ligand expression, which influences the cell counts and normal immune functioning of CD4^+^ T cells, thereby impacting the immune reconstitution in PLWH. To test this hypothesis, we plan to conduct further research, building on our previous work and utilizing the 66 clinical samples from different cohorts of PLWH, for which our team has already established a substantial resource base.

## Methods

### Ethical statement

All participants were recruited from the Beijing Primo clinical cohort, which was conducted from 2011 to 2012. Each participant provided written informed consent to participate in this study. The study, along with other related experiments, received approval from the Research Ethics Committee of Beijing Youan Hospital (approval No. 2017-13). Informed written consent was obtained in accordance with the Declaration of Helsinki, and the study was carried out in strict adherence to the approved guidelines and regulations.

### Study participants

This research is a retrospective study involving 66 PLWH from the Primo cohort of men who have sex with men (MSM) (23). Among these participants, 32 had poor CD4^+^ T-cell levels (<500 cells/μL after 4 years of ART) and were classified as INRs, while 34 exhibited immunological recovery (>500 cells/μL after 4 years of ART) and were classified as IRs. Blood specimens were collected from the PLWH both before the initiation of ART and after 4 years of treatment. Additionally, 35 healthy controls (HCs) of similar age to the PLWH were included in the study. These HCs were volunteers from the MSM high-risk negative cohort at the Clinical and Research Center for Infectious Diseases, Beijing Youan Hospital, and were matched for age and gender with the PLWH. All participants were divided into five groups: HCs, pre-ART INR (INR pre-ART), post-ART INR (INR-ART), pre-ART IR (IR pre-ART), and post-ART IR (IR-ART). In this study, INRs were defined as individuals whose viral load remained undetectable for more than 1 year after ART but whose CD4^+^ T-cell count remained below 500 cells/μL at 4 years of ART. IRs were defined as individuals with a CD4^+^ T-cell count exceeding 500 cells/μL. The baseline CD4^+^ T-cell levels for both INRs and IRs at the time of enrollment were approximately 200 cells/μL.

### The flow cytometry protocol

After collecting venous blood from the subjects, peripheral blood mononuclear cells (PBMCs) were isolated using lymphocyte separation medium according to the protocol, and flow cytometry was employed for analysis.

NK cell subsets and surface receptor staining. NK cells and CD4^+^ T cells were stained using anti-CD3-pecy7, anti-CD16-APC, anti-CD56-apccy7, anti-CD57-percp, anti-CD69-FITC, anti-CD8-precp, anti-CD4-pecy7 to stain the receptors of NK cells and CD4^+^ T cells. CD4^+^ T-cell subsets, CD4^+^ T activation, and depletion staining. CD4^+^ T cells were stained using the anti-human CD3-V450, anti-human CD4-PERCP, anti-human CD8-pecy7, anti-human CCR7-PE, anti-human CD45RO-APCCY7, anti-human CD31-APC to be divided into different subsets. Staining for CD4^+^ T-cell activation was using anti-human CD3-percp, anti-human CD4-APC, anti-human CD8-FITC, anti-human CD38-PECY7, anti-human HLA-DR-PE. Staining for CD4^+^ T-cell depletion was using anti-human CD3-PECY7, anti-human CD4-percp, anti-human CD8-FITC, anti-human CD274-APC. Staining for NKG2D ligands was using anti-human MICA/B-PECY7, anti-human CD261-APCCY7, anti-human CD4-FITC, anti-human CD95(CD262)-BV421 and anti-human ULBP2-5-PE. Ligands for death receptors on NK cells were stained using anti-human CD3-percp, anti-human CD253-APC, anti-human CD16-FITC and anti-human CD178-BV421. Staining of CD4^+^ T cells for proliferation, apoptosis, and anti-apoptosis. After surface staining using anti-human CD3-PERCP and anti-human CD4-FITC, broken the cell membrane, and then the fluorescent antibodies anti-human Ki67-Pecy7, anti-human CC3-V450, anti-human BCL-2-AF647, were added for intracellular staining.

Detection was performed using the FACS Canto II flow cytometer, and data were collected using BD Canto II Diva software version 6.0. Data analysis was conducted using FlowJo V10, with gates applied based on the characteristics of the target cells. The gating strategy for flow cytometry is provided in the **Supplementary Materials**.

### Statistical analysis

Data were analyzed using Jamovi version 2.6.17 and GraphPad 6.0 software. To compare differences between experimental groups, a normality test (Kolmogorov Smirnov test) was performed on all continuous variable data. Data that were not normally distributed were expressed as medians, and comparisons between experimental groups were made using the Wilcoxon signed-rank test or the Mann-Whitney U rank-sum test. The Spearman rank correlation test was employed to determine correlations between the experimental groups. For count data that followed a normal distribution, the results were expressed as mean ± standard deviation, and comparisons between groups were conducted using the paired samples t-test. For comparisons involving multiple groups, analysis of variance (ANOVA) was used. Count data comparisons were made using the chi-square test. A *P*-value of less than 0.05 was considered to indicate statistical significance. In the figures presented in the Results section, *P < 0.05, **P < 0.01, ***P < 0.001, ****P < 0.0001.

## Results

### Clinical characteristics of subjects enrolled in this study

Samples were collected from PLWH at two time points: baseline and 4 years after the initiation of ART. Observations were categorized into five groups: IR pre-ART (n = 34), IR-ART (n = 34), INR pre-ART (n = 32), INR-ART (n = 32), and HC (n = 35). We found that no statistically significant difference was observed in plasma HIV-1 viral load before ART between the IR and INR groups. After four years of ART, all PLWH achieved viral suppression, with undetectable plasma HIV-1 viral loads. The CD4^+^ T-cell count in PLWH within the IR group was higher than that in the INR group. The change in CD4^+^ T-cell count (ΔCD4) also differed between groups. CD8^+^ T-cell counts were comparable between the IR and INR groups before ART but were higher in the IR group than in the INR group after ART. No significant differences were found in the CD4/CD8 ratio or demographic characteristics between the two groups. As a continuation of previous research, this study utilizes the same cohort as our team’s previously published study (23), with clinical characteristics detailed in **Table 1**.

**Table 1:** Basic and clinical characteristics of this study.

	Healthy controls		PLWH		*P*-value
			INRs	IRs	
Number	35		32	34	ns
Mean age (Y)	32 (26-53)		34 (21-57)	33 (23-55)	ns
		**CD4^+^ T-cell counts (cells/μL)**			
pre-ART	NA		207	227	<0.05
			(100-313)	(130-313)	
ART	NA		380	680	<0.0001
			(163-497)	(505-1240)	
ΔCD4	NA		162.5	454.5	<0.05
			(-56-377)	(248-1049)	
		**CD8^+^ T-cell counts** (**cells/μL)**			
pre-ART	NA		664.5	766.5	ns
			(255-1716)	(331-1928)	
ART	NA		570	919	<0.0001
			(229-1192)	(515-1909)	
		**CD4/CD8 T-cell ratio**			
pre-ART	NA		0.25	0.28	ns
			(0.12-0.76)	(0.11-0.67)	
ART	NA		0.62	0.7	ns
			(0.34-1.31)	(0.45-1.36)	
		**Viral load (log10, copies/mL)**			
pre-ART	NA		4.41	4.06	ns
			(1.97-5.26)	(1.70-5.97)	
ART	NA		ND	ND	

NA, not available; ns, not significant; ND, not detected.

ΔCD4, the change in CD4^+^ T-cell count.

### Increased expression of MICA/B, ULBP2-5 on CD4^+^ T cells in INRs

**Figure 1:**
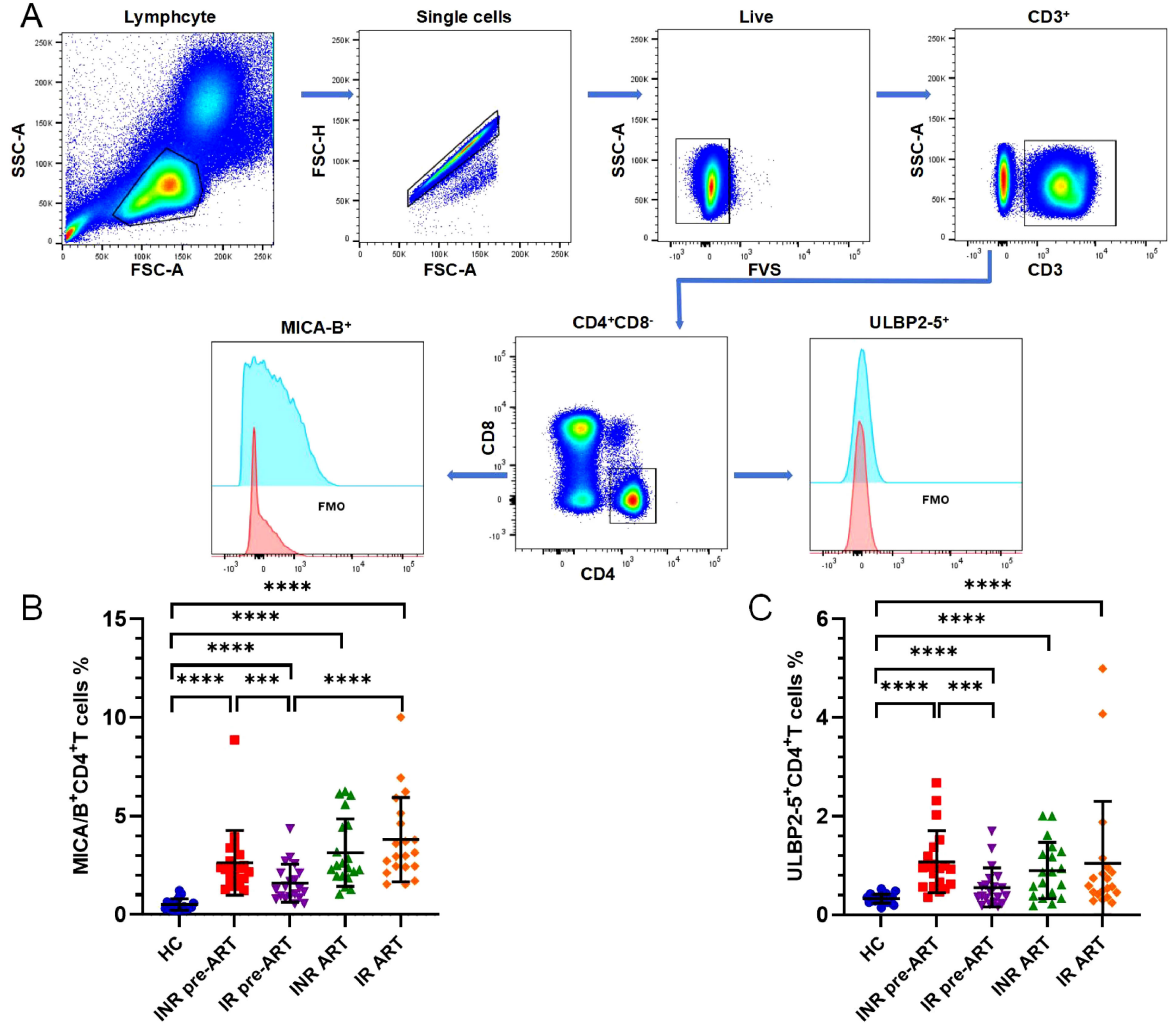
Expression of the ligands of NKG2D, MICA/B and ULBP2-5 on CD4^+^ T cells. **(A)** Flow charts of gating strategy; **(B)** Expression of MICA/B on CD4^+^ T cells; **(C)** Expression of ULBP2-5 on CD4^+^ T cells. ***P <0.001; ****P <0.0001.

Flow charts of gating strategy is displayed in **Figure 1A**. The expression of MICA/B on CD4^+^ T cells was significantly higher in both INRs and IRs before and after ART compared to HCs (**Figure 1B**). The proportion of MICA/B^+^CD4^+^ T cells was also higher in INRs than in IRs before ART, though this difference was no longer significant after ART (**Figure 1B**). Furthermore, the expression of MICA/B on CD4^+^ T cells generally increased with the duration of infection, with a more pronounced elevation observed in the IR group (**Figure 1B**). Similarly, ULBP2-5 expression on CD4^+^ T cells was elevated in PLWH compared to HCs, both before and after ART (**Figure 1C**). The proportion of ULBP2-5^+^CD4^+^ T cells was higher in INRs than in IRs before ART, with this difference becoming non-significant after ART (**Figure 1C**).

### The percentage of MICA/B^+^CD4^+^ T cells and ULBP2-5^+^CD4^+^ T cells in pre-ART PLWH exhibited a negative correlation with both CD4^+^ T-cell count and ΔCD4

**Figure 2:**
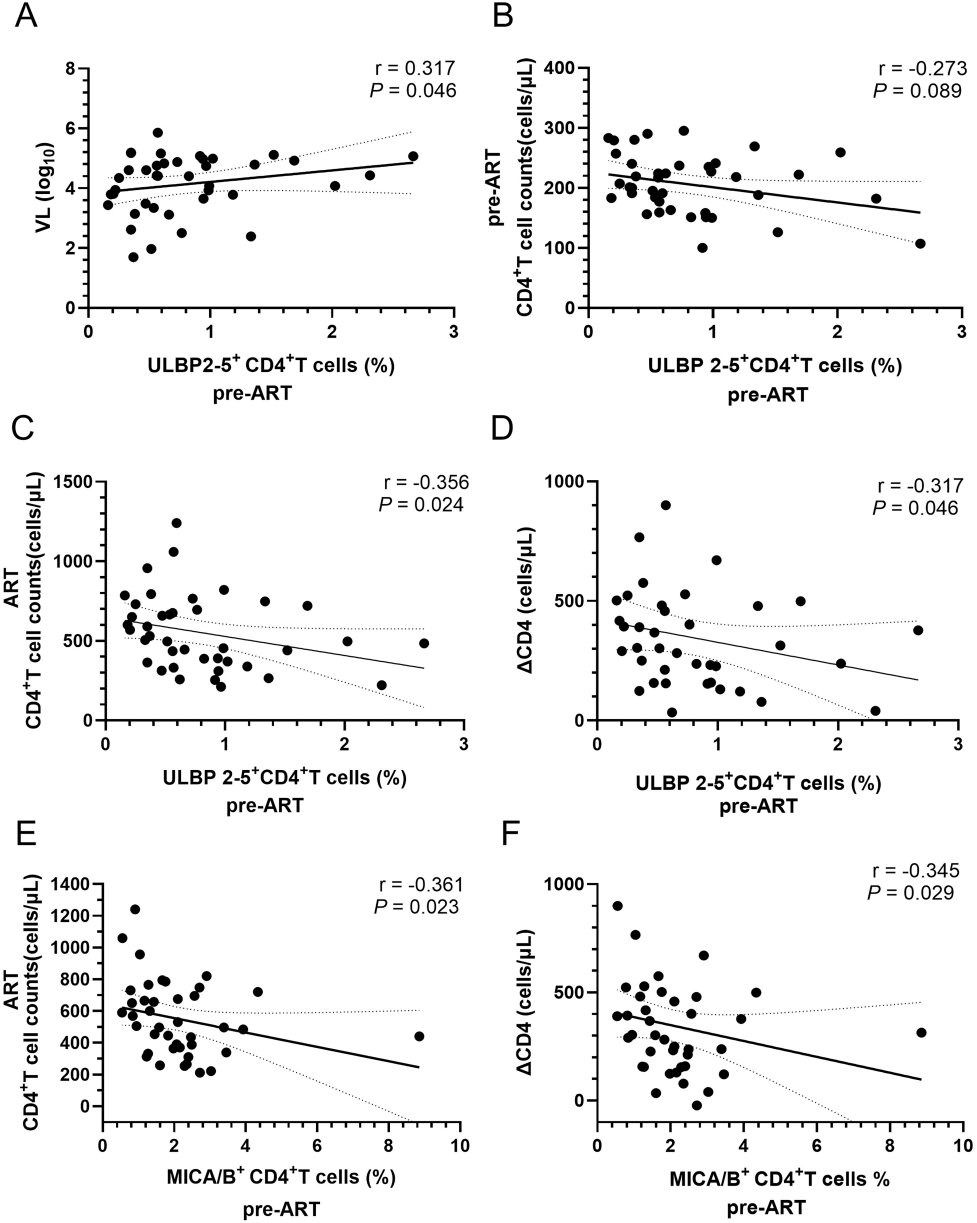
Correlation of the percentages of ULBP2-5^+^CD4^+^ and MICA/B^+^CD4^+^ T cells with CD4^+^ T-cell counts, and ΔCD4 in PLWH before ART. **(A)** Correlation of the percentage of ULBP2-5^+^CD4^+^ T cells before ART with viral load; **(B)** Correlation of the percentage of ULBP2-5^+^CD4^+^ T cells with CD4^+^ T cell counts before ART; **(C)** Correlation of pre-ART ULBP2-5^+^CD4^+^ T-cell percentage with post-ART CD4^+^ T-cell counts; **(D)** Correlation of pre-ART ULBP2-5^+^CD4^+^ T-cell percentage with ΔCD4; **(E)** Correlation of pre-ART MICA/B^+^CD4^+^ T percentage with post-ART CD4^+^ T-cell counts; **(F)** Correlation of pre-ART MICA/B^+^CD4^+^ T percentage with ΔCD4.

NKG2D is a critically activatory receptor involved in NK cell-mediated lysis of autologous activated CD4^+^ T cells. Activated CD4^+^ T cells express elevated levels of NKG2D ligands, including MIC-A, MIC-B, and ULBP2-5. The results in this study indicated that the proportion of ULBP2-5^+^CD4^+^ T cells before ART in PLWH was positively correlated with HIV viral load but negatively correlated with CD4^+^ T-cell counts pre-ART, as shown in **Figures 2A, B**. The proportion of MICA/B^+^CD4^+^ T cells and ULBP2-5^+^CD4^+^ T cells before ART in PLWH was negatively correlated with CD4^+^ T-cell count after 4 years of ART and ΔCD4, as illustrated in **Figures 2C–F**.

### The expression of CD95 on MICA/B^+^CD4^+^ T cells and ULBP2-5^+^CD4^+^ T cells was higher in pre-ART INRs compared to IRs

**Figure 3:**
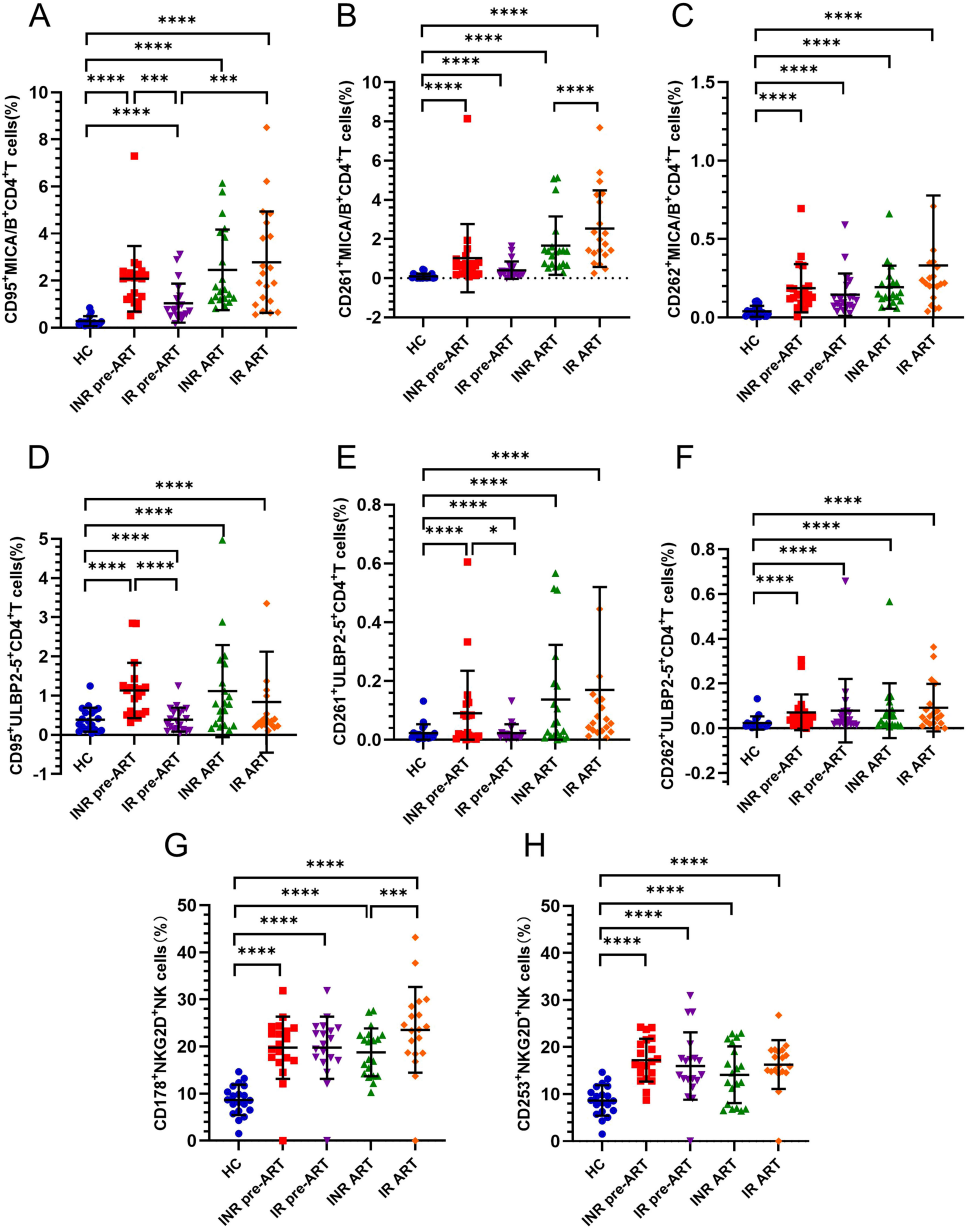
Expression of CD95, CD261, CD262 death receptors on CD4^+^ T cells and the expression of their corresponding ligands CD178, CD253 on NK cells. **(A–C)** Percentage of CD95, CD261, CD262 expression on MICA/B^+^CD4^+^ T cells; **(D–F)** Percentage of CD95, CD261, CD262 expression on ULBP2-5^+^CD4^+^ T cells; **(G–H)** Percentage of CD178, CD253 expression on NKG2D^+^NK cells. *P <0.05; ***P <0.001; ****P <0.0001.

To investigate the transmission of death receptor (DR) signals between NK cells and CD4^+^ T cells, this study analyzed the expression of death receptors CD95, CD261, and CD262 on CD4^+^ T cells, and their corresponding ligands CD178 and CD253 on NK cells. Both before and after ART, CD95 expression on MICA/B^+^CD4^+^ T cells was significantly higher in both INRs and IRs compared to HCs (**Figure 3A**). Prior to ART, CD95 expression on MICA/B^+^CD4^+^ T cells was elevated in INRs compared to IRs (**Figure 3A**). There was a gradual increase in CD95 expression on MICA/B^+^CD4^+^ T cells from baseline to 4 years of ART, with a more pronounced trend observed in IRs (**Figure 3A**). This difference between INRs and IRs was not apparent after ART.

The expression of CD261 on MICA/B^+^CD4^+^ T cells was also significantly higher in both INRs and IRs compared to HCs (**Figure 3B**). At baseline, there was no difference between INRs and IRs, but CD261 expression on MICA/B^+^CD4^+^ T cells tended to increase with prolonged infection and was higher in IRs than in INRs at the 4-year post-ART time point (IR-ART vs. INR-ART, *P* = 0.0117; **Figure 3B**). Similarly, CD262 expression on MICA/B^+^CD4^+^ T cells was significantly higher in both INRs and IRs compared to HCs (**Figure 3C**), with no notable difference between INRs and IRs.

CD95 expression on ULBP2-5^+^CD4^+^ T cells was consistently higher in INRs and IRs than in HCs, both before and after ART (**Figure 3D**). At baseline, without ART, CD95 expression on ULBP2-5^+^CD4^+^ T cells was significantly higher in INRs compared to IRs (**Figure 3D**), with this difference disappearing after ART. The expression levels of CD261 and CD262 on ULBP2-5^+^CD4^+^ T cells were generally minimal, but these markers were higher in both INRs and IRs compared to HCs before and after ART. Before ART, CD261 expression on ULBP2-5^+^CD4^+^ T cells was higher in INRs than in IRs (**Figure 3E**), whereas CD262 expression did not differ between INRs and IRs (**Figure 3F**).

No significant differences were found in the expression of CD178 on NKG2D^+^ NK cells or CD253 on NKG2D^+^ NK cells between INRs, IRs. Furthermore, CD178 on NKG2D^+^ NK cells or CD253 on NKG2D^+^ NK cells was significantly higher in both INRs and IRs compared to HCs (**Figure 3H**). CD178 expression on NKG2D^+^ NK cells was notably higher in IRs compared to INRs only at the 4-year ART time point (**Figure 3G**). Given that CD95 expression in pre-ART INRs was higher than in IRs on both MICA/B^+^CD4^+^ T cells and ULBP2-5^+^CD4^+^ T cells, we further analyzed the correlation between CD95 expression and CD4^+^ T-cell counts to assess its impact on immune reconstitution.

### Percentages of CD95 expression on MICA/B^+^CD4^+^ T cells and ULBP2-5^+^CD4^+^ T cells before ART were negatively correlated with CD4^+^ T-cell counts and ΔCD4

**Figure 4:**
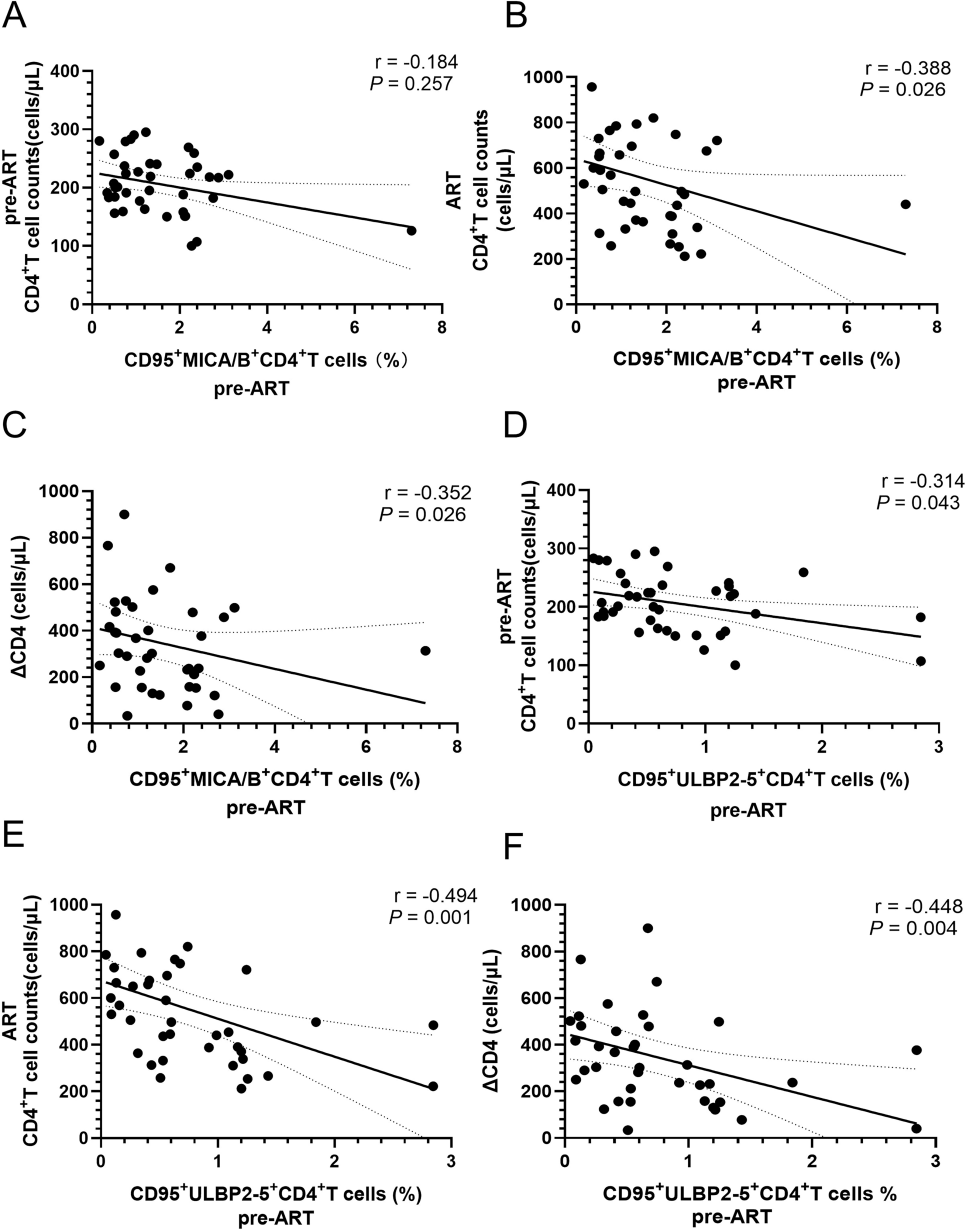
Correlations of the percentages of CD95 expression on MICA/B^+^CD4^+^ cells and ULBP2-5^+^CD4^+^ T cells before ART with the parameters of immune reconstitution in PLWH. **(A)** Correlation of the percentage of CD95 expression on MICA/B^+^CD4^+^ T cells with CD4^+^ T-cell counts before ART; **(B)** Correlation between the percentage of CD95 expression on MICA/B^+^CD4^+^ T cells before ART and CD4^+^ T-cell counts after ART; **(C)** Correlation of the percentage of CD95 expression on MICA/B^+^CD4^+^ T cells before ART with ΔCD4; **(D)** Correlation of the percentage of CD95 expression on ULBP2-5^+^CD4^+^ T cells with CD4^+^ T-cell counts before ART; **(E)** Correlation of the percentage of CD95 expression on ULBP2-5^+^CD4^+^ T cells before ART with CD4^+^ T-cell counts after four years of ART; **(F)** Correlation of the percentage of CD95 expression on ULBP2-5^+^CD4^+^ T cells before ART with the levels of ΔCD4.

CD4^+^ T-cell counts at 4 years after ART and the number of ΔCD4 were negatively correlated with the proportion of CD95 expressing on MICA/B^+^CD4^+^ T cells before ART. There was also a negative correlation with CD4^+^ T-cell counts before ART, though the *P* value does not reach statistical significance (**Figures 4A–C**). Furthermore, the levels of CD95 expression on ULBP2-5^+^CD4^+^ T-cell before ART showed a negative correlation with CD4^+^ T-cell counts before ART, CD4^+^ T-cell counts at 4 years after ART, and ΔCD4 (**Figures 4D–F**). The proportion of CD261 expression on ULBP2-5^+^CD4^+^ T cells was higher in INRs compared to IRs before ART, though this receptor overall expression was lower, and no statistically significant correlations with factors associated with immune reconstitution were found.

### CD4^+^ T cells differentiation and immune status of INRs and IRs

To elucidate the subset distribution and immune status of CD4^+^ T cells in populations exhibiting poor immune reconstitution, we conducted a comprehensive analysis focusing on various parameters. This analysis included the distribution of naive CD4^+^ T cells (CD4^+^ Tn), central memory CD4^+^ T cells (CD4^+^ Tcm), effector memory CD4^+^ T cells (CD4^+^ Tem), and terminally differentiated effector memory CD4^+^ T cells (CD4^+^ Temra), as well as markers of activation (CD38^+^HLA-DR^+^), proliferation (Ki67), and depletion (PD-L1/CD274). Additionally, we assessed apoptosis (caspase-3), anti-apoptotic mechanisms (BCL-2), and thymic output (recent thymic emigrants [RTE]) to identify and characterize differences between groups.

**Figure 5:**
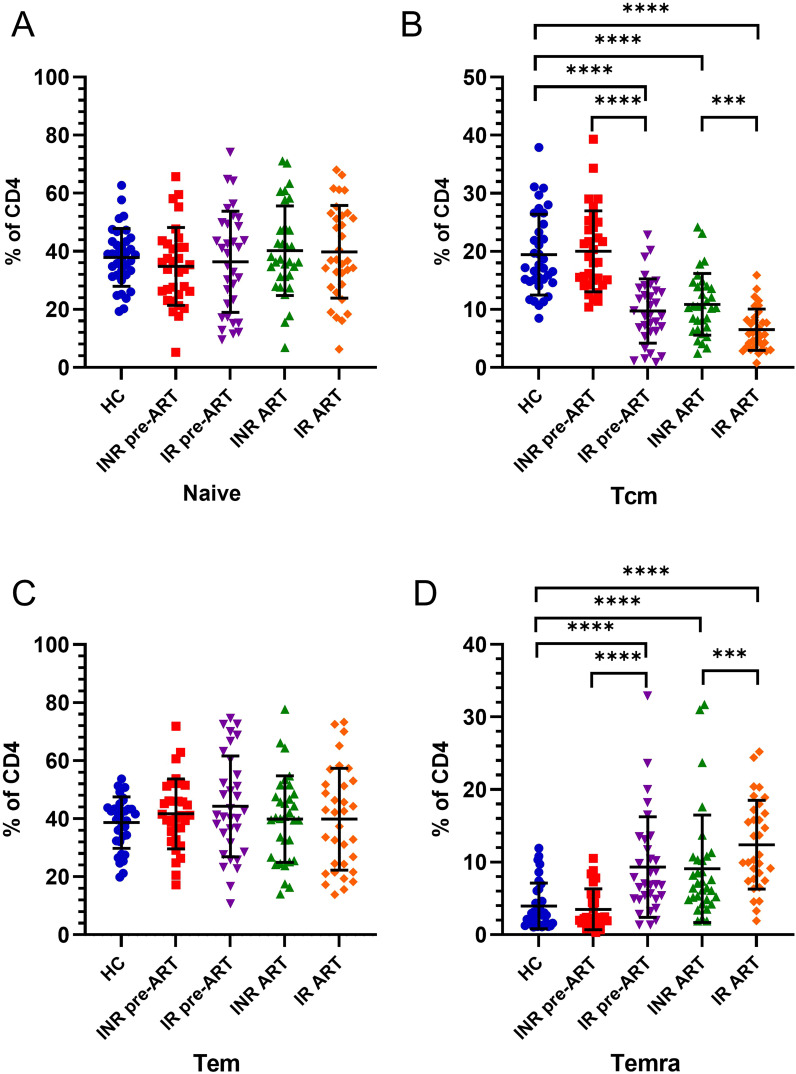
Changes in the differentiation status of CD4^+^ T cells in INRs and IRs before and after ART. **(A)** Changes in CD4^+^ Tn cells before and after ART; **(B)** Changes in CD4^+^ Tcm cells before and after ART; **(C)** Changes in CD4^+^ Tem cells before and after ART; **(D)** Changes in CD4^+^ Temra cells before and after ART. ***P <0.001; ****P <0.0001.

There were no significant differences in the proportions of CD4^+^ Tn or CD4^+^ Tem cells as a percentage of total CD4^+^ T cells between individuals with INRs and IRs. However, notable changes were observed in the CD4^+^ Tcm and CD4^+^ Temra cell subsets (**Figure 5**). After receiving ART, a general decreasing trend in the proportion of CD4^+^ Tcm cells was evident in both INRs and IRs (**Figure 5B**). Specifically, both INRs and IRs exhibited lower proportions of CD4^+^ Tcm cells compared to HCs. Notably, IRs had significantly lower proportions of CD4^+^ Tcm cells than HCs before ART, while INRs demonstrated a gradual decline, reaching lower levels compared to HCs only after 4 years of ART. The proportion of CD4^+^ Tcm cells among total CD4^+^ T cells was significantly higher in INRs compared to IRs both before and after ART. An overall increasing trend was observed in the proportion of CD4^+^ Temra cells in both INRs and IRs (**Figure 5D**). Prior to ART, the proportion of CD4^+^ Temra cells among total CD4^+^ T cells in INRs was comparable to that of HCs, whereas IRs exhibited significantly higher proportions. With prolonged infection, the proportions of CD4^+^ Temra cells were elevated in both INRs and IRs compared to HCs. Moreover, the proportion of CD4^+^ Temra cells in IRs, both before and after ART, was significantly higher than in INRs.

From the subset distribution characteristics of CD4^+^ T cells, it is evident that the reduction of CD4^+^ T cells in PLWH is primarily characterized by a loss of the CD4^+^ Tcm cell subset and an expansion of the CD4^+^ Temra cell subset. Specifically, individuals with INRs exhibit a higher proportion of CD4^+^ Tcm cells compared to IRs and a lower proportion of CD4^+^ Temra cells compared to IRs.

**Figure 6:**
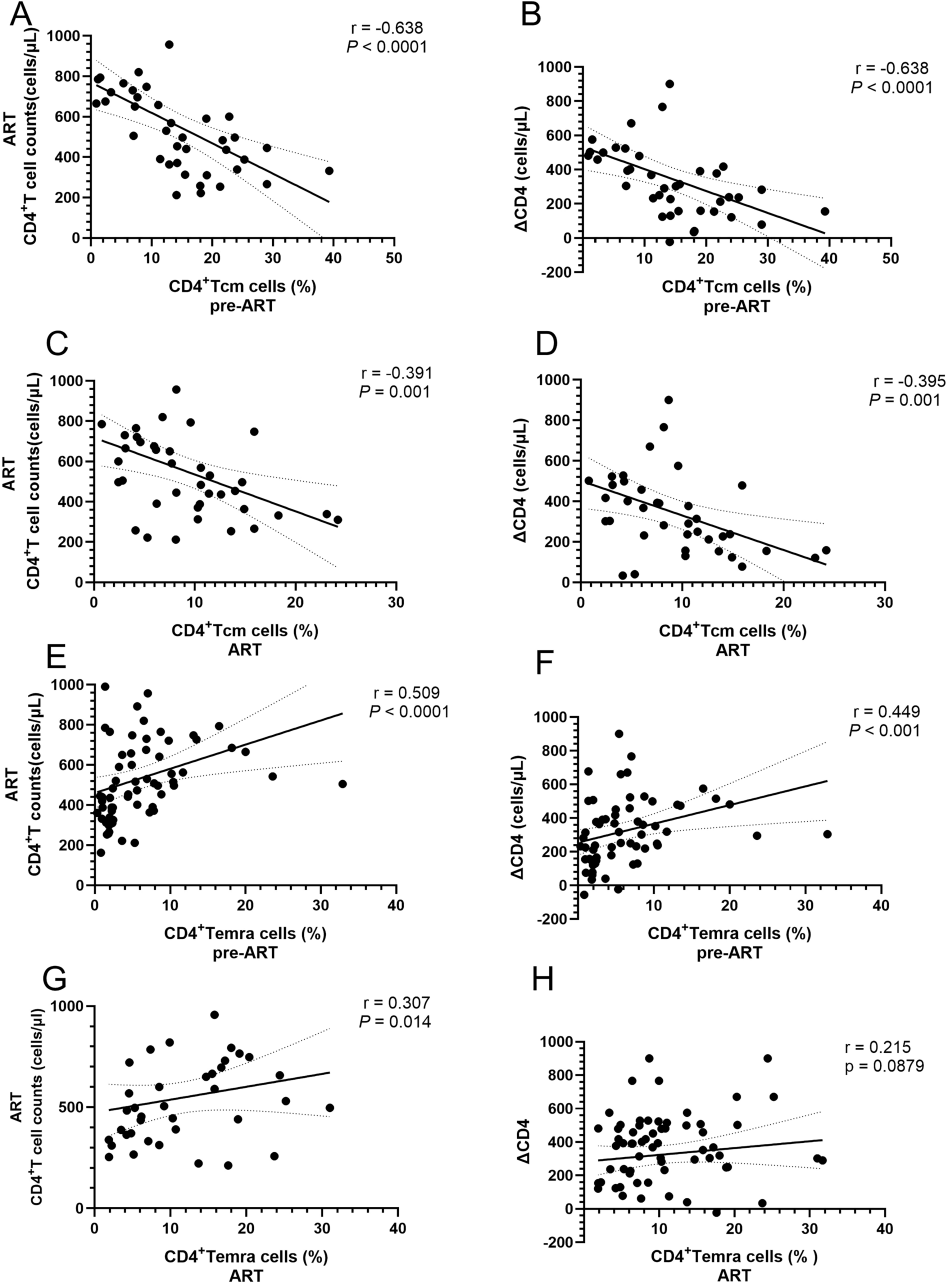
Correlations between the proportions of CD4^+^ T-cell subsets and CD4^+^ T-cell counts in PLWH. **(A)** Correlation between the proportion of CD4^+^ Tcm cells before ART and CD4^+^ T-cell counts after 4 years of ART; **(B)** Correlation between the proportion of CD4^+^ Tcm cells before ART and the levels of ΔCD4; **(C)** Correlation between the proportion of CD4^+^ Tcm cells and CD4^+^ T-cell counts after ART; **(D)** Correlation between the proportion of CD4^+^ Tcm cells after ART and ΔCD4; **(E)** Correlation between the proportion of CD4^+^ Temra cells before ART and CD4^+^ T-cell counts after 4 years of ART; **(F)** Correlation between the proportion of CD4^+^ Temra cells before ART and the levels of ΔCD4; **(G)** Correlation between the proportion of CD4^+^ Temra cells and CD4^+^ T-cell counts after ART; **(H)** Correlation between the proportion of correlation between the proportion of CD4^+^ Temra cells after ART and the levels of ΔCD4.

To understand how these subset proportion disturbances affect CD4^+^ T-cell counts, we conducted a correlation analysis. The results indicated that the proportion of CD4^+^ Tcm cells before ART was significantly inversely correlated with CD4^+^ T-cell counts after ART and ΔCD4 (**Figures 6A, B**). Similarly, the proportion of CD4^+^ Tcm cells after ART also showed a significant negative correlation with CD4^+^ T-cell counts after ART and ΔCD4 (**Figures 6C, D**). In contrast, the proportion of CD4^+^ Temra cells, unlike CD4^+^ Tcm cells, was positively correlated with CD4^+^ T-cell counts after ART and ΔCD4 before ART (**Figures 6E, F**). After ART, the proportion of CD4^+^ Temra cells continued to show a positive correlation with CD4^+^ T-cell counts after ART (**Figure 6G**) and exhibited a positive trend with ΔCD4, although this trend did not achieve statistical significance (**Figure 6H**). In contrast, no significant positive correlations were found between the CD4^+^ Tn cell subset or the CD4^+^ Tem cell subset and CD4^+^ T-cell counts.

### Activation, proliferation, exhaustion, apoptosis, anti-apoptosis, and thymic output capacity of CD4^+^ T cells in INRs and IRs

To elucidate the distribution and dynamic changes of CD4^+^ T-cell subsets and their immune characteristics in populations with poor immune reconstitution, we investigated various parameters including activation (CD38 and HLA-DR co-expression), proliferation (Ki67), exhaustion (PD-L1/CD274), apoptosis (caspase-3), anti-apoptosis (BCL-2), and thymic output (CD31) in CD4^+^ T cells across INRs and IRs.

**Figure 7:**
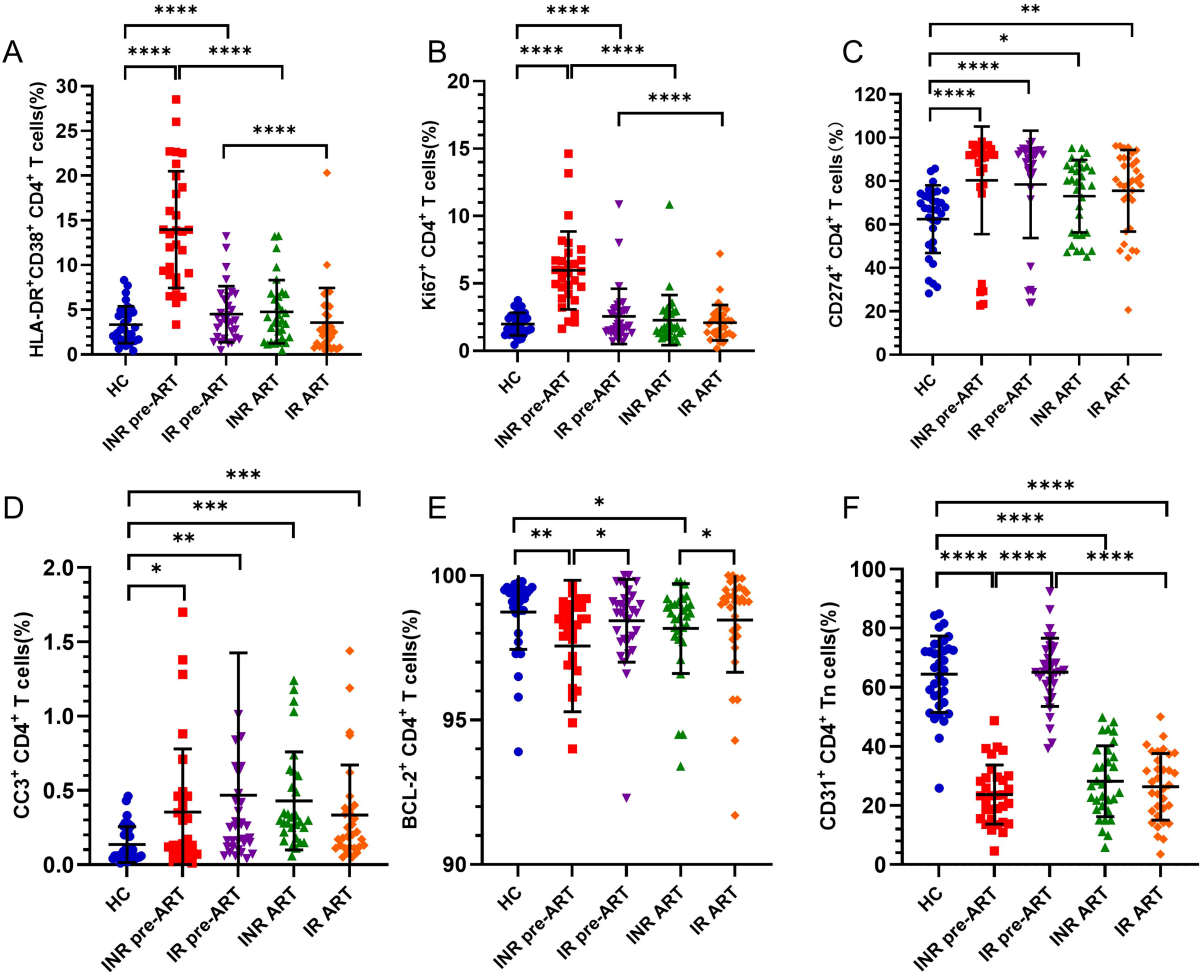
Activation, proliferation, depletion, apoptosis, anti-apoptosis, and thymic output of CD4^+^ T cells from INRs and IRs. **(A)** Percentage of CD38^+^HLA-DR^+^CD4^+^ T cells (cell activation); **(B)** Percentage of Ki67^+^CD4^+^ T cells (cell proliferation); **(C)** Percentage of CD274^+^CD4^+^ T cells (cell exhaustion); **(D)** Percentage of CC-3^+^CD4^+^ T cells (apoptosis); **(E)** Percentage of BCL-2^+^CD4^+^ T cells (anti-apoptosis) percentage; **(F)** percentage of thymus exporting CD4^+^ T cells (RTE). *P < 0.05, **P < 0.01, ***P < 0.001, ****P < 0.0001.

The activation level of CD4^+^ T cells was assessed using CD38 and HLA-DR co-expression. Prior to ART, CD4^+^ T-cell activation was significantly elevated in both INRs and IRs compared to HCs (**Figure 7A**). Following ART, activation levels in both INRs and IRs decreased to near-normal levels.

The proportion of proliferating Ki67^+^CD4^+^ T cells was also significantly higher before ART in both INRs and IRs compared to HCs (**Figure 7B**). Over time, CD4^+^ T-cell proliferation in both INRs and IRs gradually decreased to levels comparable to those in HCs.

PD-L1 (CD274) expression, a marker of CD4^+^ T-cell exhaustion, was significantly elevated in both INRs and IRs compared to HCs, with no significant difference between INRs and IRs (**Figure 7C**).

Caspase-3 expression, indicative of apoptosis, was significantly higher in both INRs and IRs compared to HCs, both before and after ART (**Figure 7D**).

BCL-2, an anti-apoptotic protein, was lower in INRs compared to HCs and IRs both before and after ART. In contrast, BCL-2 expression in IRs did not differ significantly from that in HCs (**Figure 7E**).

The proportion of CD31^+^CD4^+^ Tn cells, representing RTEs, was significantly lower in INRs compared to HCs before ART. In contrast, the proportion of RTE cells in IRs did not differ from HCs and was significantly higher than in INRs before ART. Despite ART, the thymic output capacity in INRs did not improve, remaining lower than in HCs. The proportion of RTE cells in IRs decreased significantly over time, remaining significantly lower than in HCs (**Figure 7F**).

In summary, before ART, CD4^+^ T-cell activation, proliferation, depletion, and apoptosis levels were elevated in both INRs and IRs compared to HCs. Post-treatment, activation and proliferation levels returned to normal, while apoptosis and depletion levels remained elevated. Furthermore, the anti-apoptotic capacity in INRs and IRs was lower than that in HCs, with INRs exhibiting lower levels compared to IRs. Thymic output capacity was significantly impaired in INRs before treatment and remained low after ART, whereas IRs showed no significant differences from HCs before ART, though their thymic output capacity decreased over time.

## Discussion

This study aimed to elucidate the relationship between the imbalance in NKG2D receptors/ligands expression and immune reconstitution in HIV-1 infection. Additionally, the study characterized CD4^+^ T-cell subsets and their functional status in the context of impaired immune reconstitution.

The ligands of NKG2D, specifically MICA/B and ULBPs, have garnered considerable attention in immunotherapeutic strategies aimed at enhancing NK cell function. The viral proteins of HIV-1 influence the expression of MICA/B, ULBPs on target cells, thereby sensitizing them to NK cell-mediated cytotoxicity (25). Notably, the viral proteins of HIV-1 impact the expression of these ligands on target cells, affecting their susceptibility to NK cell-mediated killing. Our previous research showed that after ART, the expression of NKG2D on CD56^dim^CD16^dim/-^ NK cell subsets significantly increased in IR and INR, and the former was significantly higher than the latter (23). Consequently, our current study focused on the expression of NKG2D ligands (MICA/B and ULBPs) on CD4^+^ T cells.

Our findings reveal that the expression of MICA/B and ULBP2-5 on CD4^+^ T cells was significantly higher in INRs and IRs compared to HCs, both before and after antiviral treatment. The proportions of MICA/B^+^ CD4^+^ T cells and ULBP2-5^+^CD4^+^ T cells were higher in INRs than in IRs before antiviral treatment; however, this difference diminished post-treatment. The expression levels of MICA/B and ULBP2-5 on CD4^+^ T cells in INRs before antiviral treatment were negatively correlated with CD4^+^ T-cell counts and ΔCD4 cells. Our study demonstrates that in a cohort with poor immune reconstitution, INRs exhibit elevated expression of NKG2D ligands MICA/B and ULBP2-5 on CD4^+^ T cells, which contributes to increased sensitivity of CD4^+^ T cells to NKG2D^+^ NK cell-mediated killing and hampers CD4^+^ T-cell recovery.

CD95 expression was elevated on MICA/B^+^CD4^+^ T cells and ULBP2-5^+^CD4^+^ T cells in INRs compared to IRs. Correlation analyses indicated that the percentage of baseline CD95^+^ULBP2-5^+^CD4^+^ T cells and CD95^+^MICA/B^+^CD4^+^ T cells were inversely correlated with ΔCD4 and CD4^+^ T-cell counts after four years. These results suggest that CD95 expression on MICA/B^+^ and ULBP2-5^+^CD4^+^ T cells may hinder CD4^+^ T-cell recovery. NK cells also modulate adaptive immune responses and negatively regulate T cells through upregulation of ligand-mediated killing of CD4^+^ T cells by NKG2D ligands (26, 27). Additionally, NK cells induce CD4^+^ T-cell apoptosis in a TRAIL-dependent manner. Although CD261 expression was upregulated in MICA/B^+^CD4^+^ T cells, no correlation with CD4^+^ T-cell counts was found. Thus, the mechanism of NKG2D-dependent NK cell-mediated CD4^+^ T-cell apoptosis warrants further investigation.

Therapeutic approaches targeting NK cell ligands represent a novel and promising strategy to improve immune reconstitution in PLWH, particularly among INRs who experience poor CD4^+^ T-cell recovery despite effective ART. One potential strategy involves blocking the interaction between NK cell receptors and their ligands (12). Specifically, inhibiting the binding of NKG2D to its ligands, including MICA/B and ULBP2-5, could reduce NK cell-mediated lysis of CD4^+^ T cells and protect these cells from excessive depletion. This could be achieved through the use of monoclonal antibodies or small-molecule inhibitors targeting the NKG2D pathway (28–30). Additionally, modulating the aberrant expression of these ligands on CD4^+^ T cells, for example, through RNA interference or CRISPR/Cas9-based gene editing, may further reduce their vulnerability to NK cell-mediated killing and promote a more robust immune recovery. Another promising avenue involves enhancing regulatory NK cell functions to restore immune balance. By increasing the activity of inhibitory NK cell receptors or reducing activatory receptor signaling, such as that mediated by NKG2D, it may be possible to mitigate the cytotoxic effects of NK cells on CD4^+^ T cells while preserving their essential roles in immune surveillance (31, 32). This approach has the potential to protect critical immune cells and promote overall immune homeostasis in PLWH. Combining NK ligand-targeted therapies with ART could provide synergistic effects by simultaneously addressing viral suppression and immune dysregulation. Such an integrated approach may enhance both CD4^+^ T-cell recovery and immune function, offering improved outcomes for INRs. To achieve this, preclinical studies using models such as humanized mice will be critical for evaluating the efficacy and safety of these therapies. Promising findings from preclinical research could then be translated into clinical trials to assess their therapeutic potential in INRs.

By characterizing the distribution of CD4^+^ T-cell subsets, we observed that in PLWH, the reduction in CD4^+^ T cells was predominantly characterized by a loss of the CD4^+^ Tcm cell subset and an expansion of the CD4^+^ Temra cell subset, with these changes being more pronounced in INRs. ART restored the number of CD4^+^ Tn cells, CD4^+^ Tem cells, and CD4^+^ Temra cells, but did not restore the CD4^+^ Tcm cell subset. Particularly in INRs, there was a continuous decline in CD4^+^ Tcm cells, and the loss of this subset was negatively correlated with both CD4^+^ T-cell counts and ΔCD4.

It has been established that untreated HIV-1 infection primarily targets and damages CD4^+^ central memory T cells. Although ART partially restores these alterations, immune abnormalities induced by HIV-1 infection persist (33, 34). PLWH exhibit a decrease in Tn cells and an increase in functional T lymphocyte subsets within peripheral blood (35–37). In the immune microenvironment, initial T cells can differentiate into various functional subsets under the influence of cytokines, growth factors, and hormones, with different subset contributing to the antiviral immune response. CD4^+^ Tcm cells are crucial for the reduction or maintenance of CD4^+^ T cells following HIV infection (33, 38, 39). Several studies have shown that CD4^+^ Tcm cells are long-lived and that their stable maintenance is essential for adequate immunity. During HIV-1 infection, persistent immune activation drives accelerated cell proliferation and apoptosis, resulting in short-lived cells. Consequently, the number of regenerating CD4^+^ Tcm cells progressively decreases with the duration of infection, leading to an imbalance in homeostasis and impaired immune function, which can result in opportunistic infections. These findings suggest that early interventions aimed at preserving the regenerative capacity of CD4^+^ Tcm cells could improve CD4^+^ T-cell resilience and enhance long-term immune function.

Research indicates that INRs exhibit significantly reduced levels of peripheral blood CD34^+^ HPCs, mature lymphocyte subsets (CD8^+^ T cells, NK cells, B cells), and circulating CD34^+^ HPCs, which positively correlate with CD4^+^ T-cell counts. These reductions likely result from bone marrow dysfunction (40). While ART facilitates gradual recovery of CD4^+^ Tn cells and increases thymic output in most PLWH, INRs demonstrate significantly lower frequencies and absolute numbers of RTEs compared to IRs, reflecting impaired thymic output (41, 42). Chronic HIV infection disrupts thymic architecture, reduces CD4^+^ T-cell production, and induces peripheral T-cell depletion (43, 44). Aberrant immune activation, characterized by increased expression of activation markers (CD38^+^HLA-DR^+^) and co-inhibitory receptors (PD-1, CTLA-4, TIGIT, Tim-3), exacerbates CD4^+^ T-cell depletion and immune exhaustion (45). Our findings reveal that before ART, INRs and IRs exhibit elevated CD4^+^ T-cell activation, proliferation, depletion, and apoptosis, along with reduced anti-apoptotic and thymic output capacities. These abnormalities persist post-ART in INRs, particularly elevated apoptosis, impaired RTE production, and CD4^+^ Tcm cell imbalance, hindering immune reconstitution.

These findings illustrate that while ART effectively suppresses viral replication, it does not fully restore immune function in INRs. Persistent abnormalities, including impaired immune functions, elevated expression of NK cell ligands, and imbalances in CD4^+^ T-cell subsets, contribute to poor immune reconstitution in this subgroup. These results underscore the urgent need for adjunctive therapies targeting these mechanisms to improve clinical outcomes for INRs.

This study provides significant insights into the mechanisms underlying impaired immune reconstitution in PLWH, particularly focusing on NK cell ligands and CD4^+^ T-cell dynamics. However, several limitations and potential biases must be acknowledged, especially when interpreting the findings within the broader context of HIV research.

The study population was limited to MSM from a single cohort in Beijing, restricting the generalizability of the results to other populations, such as women, heterosexual individuals, and people from different ethnic or geographical backgrounds. Furthermore, the relatively small sample size of 66 participants reduces the statistical power of the analyses, increasing the risk of type II errors and potentially obscuring smaller but clinically meaningful effects.

Future research should investigate the molecular mechanisms underlying thymic output impairment, including the effects of persistent immune activation on thymocyte development and stromal cell interactions. Longitudinal studies with biomarkers like T-cell receptor excision circles (TRECs) are needed to assess recovery dynamics and identify critical intervention periods. Promising therapeutic strategies include IL-7 administration, thymic hormone analogs, or regenerative approaches like thymus bioengineering, though their feasibility requires further evaluation. Predictive models based on thymic output markers could enable tailored interventions for INRs. Addressing these gaps will advance our understanding of immune reconstitution and inform novel therapies to enhance recovery in PLWH with compromised immune function.

The study’s reliance on two discrete time points (pre-ART and post-ART) for data collection limits the ability to capture the dynamic progression of immune reconstitution. A longitudinal approach with multiple intermediate time points would provide a more detailed understanding of the temporal changes in immune parameters, offering a clearer view of the trajectory of immune recovery.

In conclusion, while the study makes valuable contributions to understanding the challenges of immune reconstitution in PLWH, addressing these limitations in future research will be critical. Expanding the study population, incorporating longitudinal designs, integrating functional experiments, and situating the findings within the broader research landscape will enhance the robustness and translational potential of subsequent studies.

## Conclusion

Poor immune reconstitution remains a challenging issue in HIV-1 infection, and no single factor fully elucidates its mechanisms. The role of NK cells as intermediaries between innate and adaptive immunity is crucial in regulating T-cell responses. From the perspective of NK cells, the ligands for NKG2D, namely MICA/B and ULBP2-5, were found to be upregulated on CD4^+^ T cells in patients experiencing INRs. Elevated apoptosis receptor CD95 in CD4^+^ T cells expressing NKG2D ligand in INR patients is relevant for the poor immune reconstitution pieces. This upregulation renders the CD4^+^ T cells more susceptible to NK cell-mediated cytotoxicity. The regenerative capacity of CD4^+^ Tcm cells has the potential to enhance the immune reconstitution of CD4^+^ T cells, ensuring more effective and sustained immune function. Further mechanistic studies are warranted to understand this phenomenon. Activation, proliferation, depletion, apoptosis, anti-apoptosis, and thymic output capacity of CD4^+^ T cells in INRs all show abnormal functional performance.

## Data Availability

The original contributions presented in the study are included in the article/**Supplementary Material**, further inquiries can be directed to the corresponding author/s.
