# Supplementary material for: Impaired immune reconstitution in HIV infection: the role of CD4+ T-cell-associated NKG2D ligands, CD4+ T-cell subsets imbalance, and immune function deficiency
Source: Front Immunol. 2025 Feb 21;16:1541574. doi: 10.3389/fimmu.2025.1541574 (PMC11885256; doi:10.3389/fimmu.2025.1541574)
Supplement: Supplementary file 1 [file Image1.pdf]

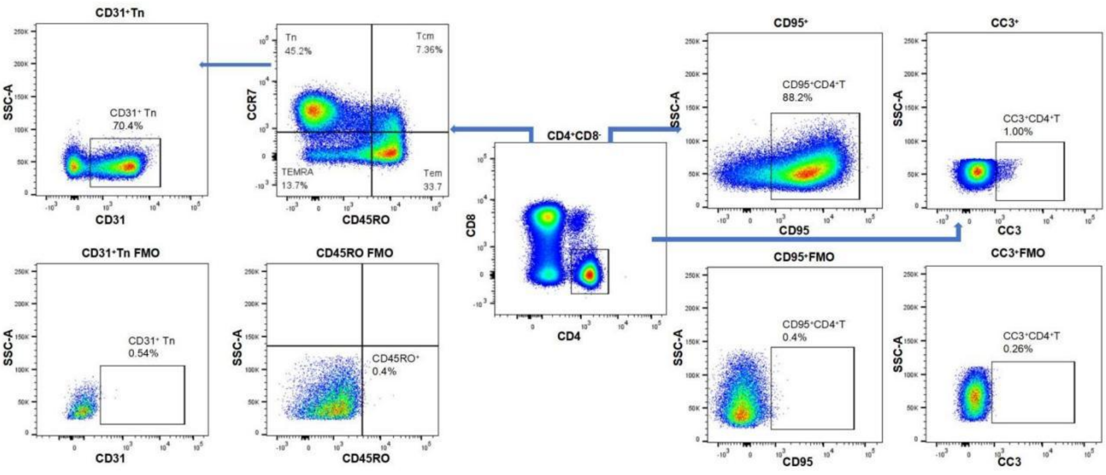

Figure S1.A Flow cytometry Gating Strategy for CD4$^+$ T Cell Subpopulations

Tn, Tcm, Tem, and Temra subsets were defined from CD4$^+$ T cell subsets based on the expression of CD45RO and CCR7; the expression of each receptor is circled separately, where CD45RO, CD31, CD95, and Caspase-3 gating were set according to the fluorescence minus one (FMO) control.

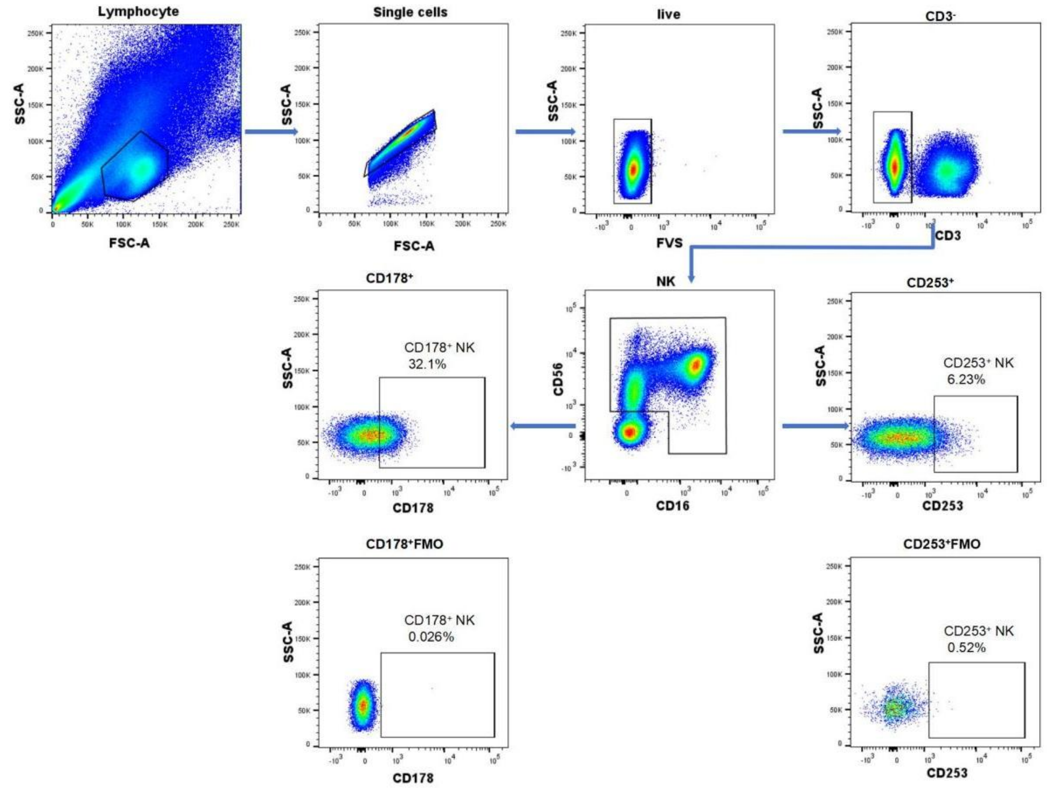

Figure S2.A Flow cytometry Gating Strategy for NK Cell Subpopulations.
Forward angle (FSC-A) and sideways angle scatter (SSC-A) for gating lymphocytes; forward angle scatter height and area circled individual cells; dead-viable staining removed dead cells circled out viable cells; followed by circling CD3-lymphocytes; NK cells were defined from CD3-negative gating based on expression of CD16 and CD56; and followed by circling expression of each receptor separately. Expression of each receptor CD178, CD253 was circled from NK cells, where CD178, CD253 gating was set according to fluorescence minus one (FMO) control.
